# The epidemiology of hepatitis B virus infection in HIV-infected and HIV-uninfected pregnant women in the Western Cape, South Africa^☆^

**DOI:** 10.1016/j.vaccine.2013.08.028

**Published:** 2013-11-12

**Authors:** M.I. Andersson, T.G. Maponga, S. Ijaz, J. Barnes, G.B. Theron, S.A. Meredith, W. Preiser, R.S. Tedder

**Affiliations:** a Division of Medical Virology, University of Stellenbosch/National Health Laboratory Service, Tygerberg, South Africa; b Blood Borne Virus Unit, Virus Reference Department, Public Health England, Colindale, London, UK; c Department of Community Health, University of Stellenbosch, South Africa; d Department of Obstetrics and Gynaecology, University of Stellenbosch and Tygerberg Hospital, South Africa; e Department of Pharmacology, University of Cape Town, South Africa; f Division of Infection and Immunity, University College London, UK

**Keywords:** HBV, HIV, Antenatal, Virus escape, Sub-Saharan Africa

## Abstract

**Objectives:**

Persistent hepatitis B virus (HBV) infection is a major cause of morbidity and mortality in sub-Saharan Africa. The HIV epidemic has the potential to affect its biology. Immunisation protocols established in the pre-HIV era are based upon data showing predominantly horizontal infant transmission. This study aimed to determine whether HIV co-infection will change the epidemiology of HBV both by increasing infectivity and by favouring the escape of viruses bearing phenotypically altered HBsAg.

**Methods:**

This retrospective cross-sectional study used antenatal samples from the 2008 Antenatal Sentinel HIV and Syphilis Prevalence Survey in the Western Cape, South Africa. All HIV-infected women were age and race-matched to HIV-uninfected women. Samples were tested for serological markers of HBV and HDV infection. HBV viral load, consensus sequencing and genotyping were performed. Luminex technology was used to determine HBsAg phenotype. All samples from HIV-infected women were tested for traces of antiretroviral drugs by mass spectrometry.

**Results:**

This study showed a trend toward loss of immune control of HBV in HIV-infected women with 3.4% of samples containing HBsAg, 18.9% contained HBeAg. In contrast, 2.9% of samples from HIV-uninfected women contained HBsAg and 17.1% of these HBeAg. The median HBV load in the HIV-infected group was 9.72 × 10^7^ IU/ml and in the HIV-uninfected group 1.19 × 10^6^ IU/ml. Genotyping showed 63/68 samples belonged to genotype A and the remainder genotype D. Mutations in the precore region were found in 35% and 33% of samples from HIV-infected and HIV-uninfected respectively. Although no major epitope ablation was found, marked variation in HBsAg profiles in HIV-infected group was demonstrated. No HDV infection was detected.

**Conclusion:**

HIV-HBV co-infected women exhibit a degree of immune escape. One in six HBV-infected pregnant women, irrespective of HIV status is HBeAg seropositive. HBV immunization of newborns in sub-Saharan Africa should be implemented.

## Highlights

• HIV-infected pregnant women have evidence of HBV escape compared to uninfected women.
• One in six HBV-infected pregnant women is HBeAg seropositive, regardless of HIV status.
• These data support the call to implement a birth dose of HBV vaccine.

## 1 Introduction

Chronic hepatitis B virus (HBV) infection is a major cause of morbidity and mortality in sub-Saharan Africa (SSA) [1] despite the availability for the last three decades of a safe and effective vaccine. Various parameters of HBV infection are altered by the immunosuppression caused by human immunodeficiency virus (HIV) infection. Those persons co-infected have a greater prevalence of HBV e antigen (HBeAg) which is a marker for infectivity [2], higher HBV DNA levels [3], more frequent HBV reactivation [4] and a higher prevalence of occult HBV infection [5]. Two resulting aspects of particular concern are higher infectivity facilitating HBV onward transmission and the potential for generation of immune escape variants during reactivation. The nature of HBV infection in pregnant women is important as it determines the risk of transmission of HBV to their infants *in utero* [6] and during parturition [7]. Despite this important relationship there are few data describing the viral parameters of HBV infection in HIV co-infected pregnant women.

HBV immunisation protocols for the prevention of infection in early life in many African countries are based on data from the pre-HIV era which indicated that HBV infection occurred predominantly horizontally between siblings and play-mates rather than vertically from mother to child [8,9]. These protocols are therefore aimed at preventing horizontal transmission during childhood rather than preventing maternal–infant transmission at birth. As a consequence the first dose of vaccine is given later in the postnatal period rather than as close to parturition as possible, leaving the neonate susceptible to infection from exposure at birth. Furthermore there is no antenatal screening programme for HBV in South Africa, nor in much of SSA.

The escape of HBV from a partially incapacitated host immune system is often associated with the generation of viral variants bearing changes in the HBV surface antigen (HBsAg). Transmission of these variants to infants has the potential to establish them in the general population. Phenotypic changes in HBsAg may reduce the efficacy of the current recombinant hepatitis B vaccines based on the short HBsAg transcript and promote the propagation of immune escape variants.

This study aimed to determine whether there is evidence to support the hypothesis that HIV co-infection will change the epidemiology of HBV both by increasing infectivity and by favouring the escape of viruses bearing phenotypically altered HBsAg.

## 2 Materials and methods

This retrospective, cross-sectional study used antenatal samples collected for the 2008 Antenatal Sentinel HIV and Syphilis Prevalence Survey [10] in the Western Cape Province of South Africa. All HIV-infected women from whom samples were available were randomly age- and race-matched to HIV-uninfected women. Approval was obtained from the University of Stellenbosch Health Research Ethics Committee and from the Provincial and National Departments of Health.

### 2.1 Recruitment of patients

Of the 9355 pregnant women recruited from antenatal clinics around the Western Cape for the 2008 survey, 1549 were seropositive for anti-HIV and were matched for age and race to 1550 HIV-uninfected women. Of the 3099 serum samples from these individuals, ten were haemolysed and discarded as unsuitable for testing. The remaining 3089 samples comprising 1546 HIV-uninfected and 1543 HIV-infected survey participants were included in the study. The date of HIV infection, the degree of immunosuppression and CD4 count were not available for the HIV-infected women.

### 2.2 HBV serological and molecular testing

Samples were tested for HBsAg using the Abbott AxSYM (Abbott Diagnostics, Chicago, IL). All samples initially reactive for HBsAg were confirmed by neutralisation using high titre human antibody to HBsAg (anti-HBs) in the Abbott Murex HBsAg GE34/36 immunoassay kit (Murex Biotech, Kent, England). Samples confirmed to contain HBsAg were tested for HBeAg and antibodies to HBeAg (anti-HBe) using DiaSorin ETI-EBK PLUS and ETI-AB-EBK PLUS immunoassay kits (DiaSorin, Saluggia, Italy). Testing for antibody to the hepatitis Delta Virus (anti-HDV) was performed on all available HBsAg-positive samples using ETI-AB-DELTAK-2 (DiaSorin).

HBV DNA quantification, consensus sequencing and genotyping were performed as previously described [11–13], on samples containing HBsAg. Sequence analysis was performed using DNASTAR (version 9).

Alignments of 1800 sequences representing all described genotypes/subgenotypes were obtained from either in-house generated sequences or from GenBank. The sequences were checked both visually and by Position Specific Scoring Matrix to identify intra-genotypic motifs. Amino acid alignments for wild-type consensus genotype specific sequences were created for the basal core promoter, precore, polymerase and HBsAg regions and used to identify mutations. Genotype determination was based on clustering within phylogenetic trees generated using the 1800 representative HBsAg nucleotide sequences.

### 2.3 HBsAg phenotyping

The impact of the observed amino acid changes on HBsAg antigenicity was investigated via *ex vivo* HBsAg phenotyping as previously described [14]. Briefly, Luminex technology was used to measure the interaction of plasma HBsAg with three monoclonal antibodies (mAbs) directed against different epitopes of the “a” determinant of HBsAg. The alteration of HBsAg antigenicity was defined by changes in the pattern of reactivity on the individual solid-phase monoclonal antibodies exceeding 2 SD of the mean when compared to expected wild type reactivity. Only samples from women infected with HBV genotype A whose plasma HBsAg was reactive in the phenotyping assays at a level equivalent to or greater than 50 IU/ml were included.

### 2.4 Antiretroviral drug residue testing

At the time of this study first line therapy for HIV infected pregnant women with a CD4 count less than 200 cells/mm^3^ was zidovudine plus lamivudine plus efavirenz or nevirapine. For those women with a CD4 count greater than 200 cells/mm^3^, zidovudine alone was administered. For those who failed first line therapy, a combination using lopinavir was prescribed. Tenofovir therapy was available for those patients who were known to be HBsAg positive. Lamivudine was used as a surrogate for tenofovir as all women on tenofovir were also administered lamivudine. In order to identify those women on combination antiretroviral therapy, and in particular those on HBV-active therapy, all samples from HIV-infected women were tested for traces of lamivudine, lopinavir, efavirenz and nevirapine by mass spectrometry, as described previously [15].

### 2.5 Statistical analysis

Categorical variables were described using number and percentages. Quantitative variables were expressed as a mean and standard deviation if normally distributed or median and interquartile range if not normally distributed. Pearson's chi squared test was used to determine association between independent variables, however where numbers in a cell were less than five, a Fisher's exact test was used instead. Student's *t*-test was used to examine the association between HIV status and HBV viral load and age and HBeAg status. ANOVA was used to determine if there was any association between HBsAg status and age, education level, or parity. All reported *p* values are for two-tailed tests. Data was analysed using Statistica, version 11 (StatSoft Inc., OK, USA).

## 3 Results

### 3.1 Demographics

The basic demographic data of the study cohort are shown in Table 1.

### 3.2 HBV and HDV serology

Ninety-seven samples from the 3089 studied were confirmed to contain HBsAg; the prevalence in HIV-uninfected pregnant women was 2.9% (44/1546) compared with 3.4% (53/1543) in HIV-infected pregnant women (Table 2). A significant association was found between HBsAg seropositivity and a lower educational grade (*p* = 0.03), but not between HBsAg status and HIV status (*p* = 0.404), age (*p* = 0.52) or parity (*p* = 0.27). All HBsAg-positive samples of sufficient volume (87) were tested for anti-HDV and all were negative.

### 3.3 HBeAg prevalence

Only 94 samples were of sufficient volume for HBeAg testing of which 17 contained HBeAg but no anti-HBe, 7/41 (17.1%) in the HIV-uninfected group compared with 10/53 (18.9%) in the HIV-infected group (Table 2). In the HIV-uninfected group, one sample contained neither HBeAg nor anti-HBe and one contained both antibody and antigen. In the HIV-uninfected group, the HBeAg seropositive mothers were significantly younger than the HBeAg seronegative mothers (21.7 yrs SD = 5.59 *vs.* 27.6 yrs SD = 4.74, *p* = 0.016). In contrast, there was no significant difference in age between HBeAg seropositive and seronegative women in the HIV-infected group.

### 3.4 HBV DNA levels

**Fig. 1:**
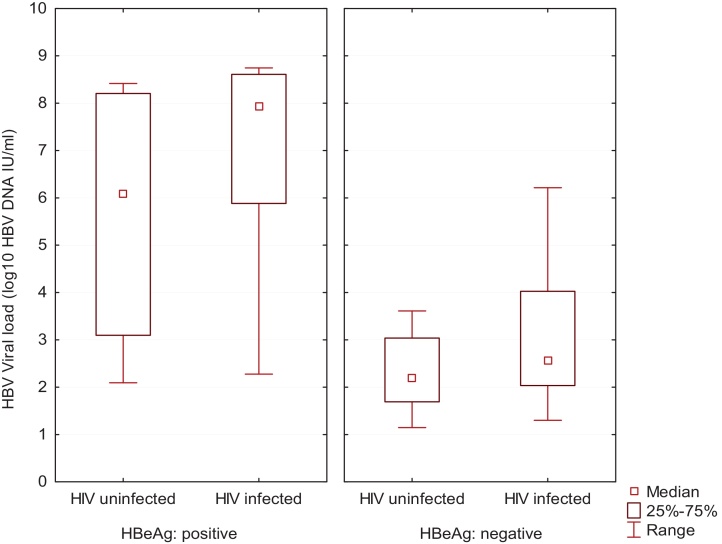
Median plasma HBV viral loads (expressed in IU/ml) according to HIV status in HBeAg positive (left) and in HBeAg negative, anti-HBe positive (right) samples displayed as box (interquartile range (IQR)) and whisker (range) plots.

In those samples from mothers who were seropositive for HBeAg, the HBV DNA was of similar range, but the median was different, in those seven not infected with HIV (120–260,000,000 IU/ml, median 1.19 × 10^6^ IU/ml) and those ten HIV-infected (190–560,000,000 IU/ml, median 9.72 × 10^7^ IU/ml) (Fig. 1 and Table 2).

The serum HBV DNA was below the level of detection in 14/75 whose serum contained anti-HBe. The proportion in whom DNA could be quantified was similar in the HIV uninfected mothers (27/32) and the co-infected mothers (34/43). Serum HBV DNA loads was of similar magnitude in the 27 HIV-uninfected women (10–140,000 IU/ml, median 108 IU/ml) and the 34 HIV-infected women (10–1,600,000 IU/ml, median 300 IU/ml) (Fig. 1 and Table 2).

### 3.5 HBV genotyping, mutational analysis and phenotyping

HBV DNA sequence and genotype analysis was successfully undertaken on 25 and 43 samples from HIV-uninfected and HIV-infected women, respectively. Phylogenetic analysis of HBsAg indicated 63 persons to be infected with virus belonging to genotype A. The remaining five persons harboured genotype D viruses; four were HIV-uninfected women and one HIV-infected. One HBV sequence from an HIV-uninfected patient bore rtV173L alone, part of the antiviral resistance profile for lamivudine; none of the 42 HBV sequences from the HIV-infected cohort carried detectable drug resistance mutations.

**Fig. 2:**
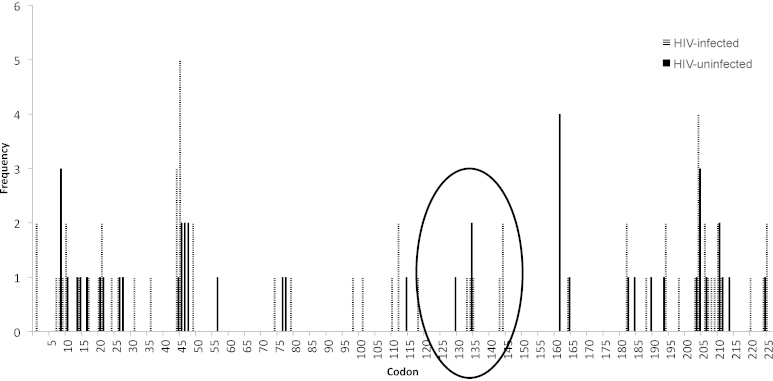
‘Hot spot’ analysis of amino acid changes across the HBsAg. Solid bars are sequences from 25 HIV-uninfected women and the hatched bars from HIV-infected women. The circled area indicates amino acid changes in the major antigenic region between codons 120 and 150.

Mapping of amino acid changes from the wild type consensus sequence was undertaken across the HBsAg region in order to identify potential mutation ‘hot spots’ (Fig. 2). Two samples from HIV-uninfected and three from HIV-infected individuals carried viruses with a number of coding changes between codons 120 and 150. One sample from an HIV-uninfected and two from HIV-infected mothers contained viruses carrying a premature stop at codon 182. The significance of changes outside codons 120–150 on HBsAg antigenicity remain unknown.

**Fig. 3:**
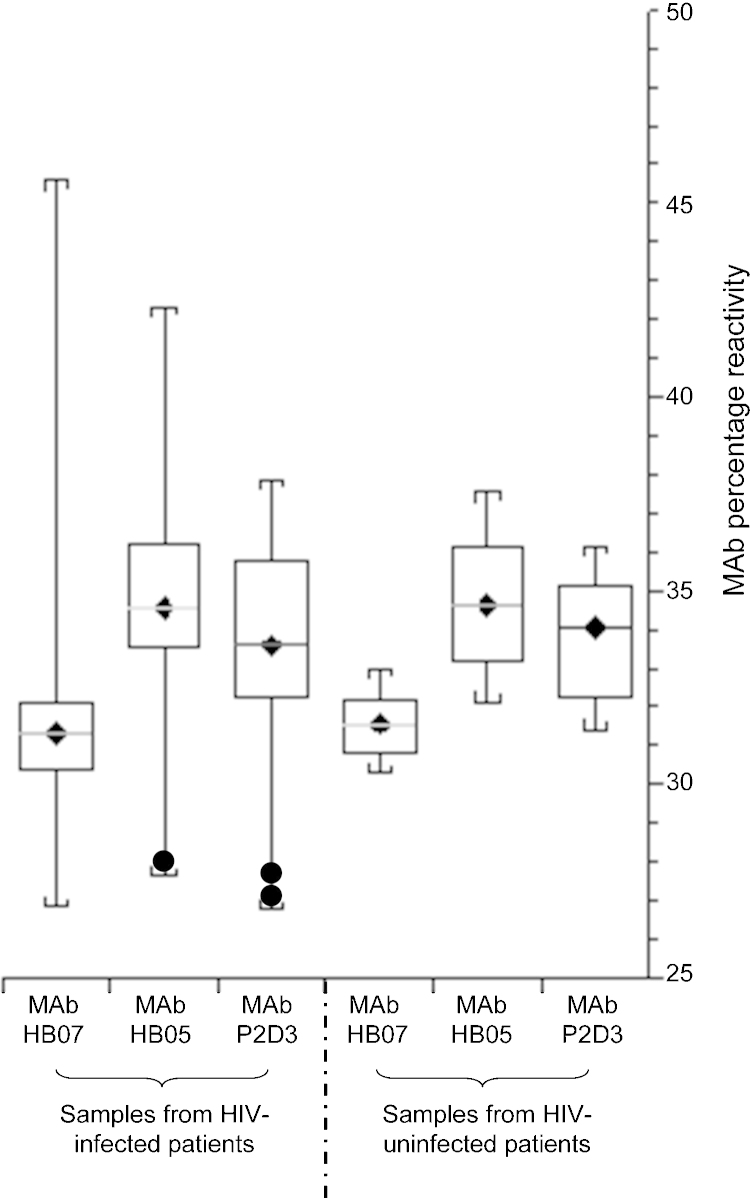
Epitope variance of plasma HBsAg expressed as box (middle quartiles) and whiskers (range) in 20 HIV-uninfected and 33 HIV-infected women. Reactivity against each monoclonal antibody solid phase is shown independently (MAb P2D3 is against a linear epitope and MAbs HB07 and HB05 are against different conformational determinants in the second loop of HBsAg) and is expressed as the percentage reactivity of each mAb as part of the total reactivity for that sample. ● denotes those samples whose reactivity fell 2 standard deviations outside the expected range for that specific MAb.

Epitope mapping analysis was undertaken in 20 HIV-uninfected and 33 HIV-infected mothers with HBV genotype A virus infection and HBsAg levels equal to or greater than 50 IU/ml. No antigen exhibited major epitope ablation. However, the variations defined by SD exceedance were clearly more marked in the surface antigen profiles in samples from HIV infected mothers (Fig. 3). Three reactions falling outside 2 SD of the mean were seen in samples from HIV-infected mothers. One virus carrying extensively mutated HBsAg sequences (M1T, R79H, L98V, M133T, P135L, D144G, D194V) and two less scarred viruses (G44E, P203G/E and S204N) had reactivities on one or more solid phases outside 2SD of the mean.

Mutations in the precore which impact on HBeAg expression were found in 33% and 35% of samples from HIV-uninfected and HIV-infected individuals respectively, with the loss of methionine at codon 1 being the most commonly observed pathway for achieving e-null viruses constitutively unable to express HBeAg. The A1762T/G1764A basal core promoter variant was observed at a similar level (38%) in samples tested from both HIV-infected and uninfected women. No significant association between HBV DNA level and the presence of these basal core promoter mutations or pre-core mutations was noted in either patient group.

### 3.6 Antiretroviral drug residue testing

Antiretroviral drug residues were detected in three of 50 HIV-HBV co-infected samples (1.5%). Lamivudine and nevirapine in two and lamivudine and lopinavir in a third sample. Three samples were of insufficient volume for testing.

## 4 Discussion

Unlike data from well-resourced countries which show a significant difference in HBsAg prevalence in HIV-infected compared to uninfected persons [16], this study confirms previous antenatal data from Africa showing little difference in the prevalence of HBsAg in HIV-infected compared with HIV-uninfected women, as found elsewhere in South Africa, Côte D’Ivoire, Malawi and Tanzania [17–20]. We have found however some evidence of loss of HBV immune control in HIV co-infected women who demonstrated a trend towards higher HBV viral loads.

In this antenatal population from the Western Cape, as many as one in six (18.1%) of HBV-infected mothers are HBeAg-seropositive and anti-HBe negative, irrespective of HIV status. This is a higher proportion than expected and contrasts with Oshitani and colleagues who found a difference in HBeAg prevalence in HIV-infected compared with HIV-uninfected women (25% *vs.* 12.3%) [21]. Our figure is lower than the high prevalence (5/12) of HBeAg positivity that we found previously in a cohort of HIV-HBV co-infected pregnant women with low CD4 counts attending a tertiary referral hospital [22]. This may reflect differences in immune competence between the two study cohorts. CD4 data was not available for the current study to confirm this suggestion.

Although an association between high HBsAg prevalence and low education was found, the reasons for this are not clear. Low educational attainment may be a surrogate for rural childhood where education is poorer than in urban areas, and HBsAg prevalence higher.

Our data also indicate that among HIV-uninfected women, those whose serum contains HBeAg are significantly younger than those who are HBeAg seronegative. This raises the question of whether HIV is having a population based effect on HBV epidemiology also in the HIV-uninfected population.

WHO guidelines recommend HBV vaccine to be administered within 24 h of delivery [23], however the first HBV vaccine dose in sub-Saharan Africa is given anytime between four and six weeks postnatally, a time appropriate to prevent horizontal transmission, but probably too late to prevent reliably perinatal transmission and the evolution of persistent HBV infection. In light of this, our observations have important implications for current hepatitis B immunisation schedules in sub-Saharan Africa where the timing of neonatal vaccine is predicated upon data generated before the HIV pandemic. This was at a time when HBeAg seropositive and, by inference, high infectivity mothers were rarely encountered in the labour wards [24]. Most mothers were therefore considered to be of low infectivity. HBV transmission to infants was deemed to occur during early childhood [8] rather than through maternal–infant transmission in spite of one baseline HBV vaccine study reporting a 10.3% HBeAg prevalence in HBV-infected females [25]. Our data clearly indicate that contrary to previous beliefs, high HBV infectivity mothers, whether defined by HBeAg seropositivity or by HBV DNA, are not uncommon in the antenatal population in South Africa and indicate that current national vaccine schedules may be inadequate to prevent mother-to-child transmission of HBV. Klingler and colleagues have produced modelling data which suggest adding a birth dose of HBV vaccine to the current regime may be cost effective [26]. This may not be as impractical as it first seems given the majority of women in SSA give birth in health care facilities [27].

Although HBV DNA load is the most important predictor of HBV transmission from mother to child [7], HBeAg remains an important marker for potential infectivity in pregnant women, particularly in those situations where viral load estimates may not be available. Not only is it associated with a high viral load, but it is also thought to cross the placenta to tolerize the neonate leading to an increased risk of chronic HBV infection [28]. HBeAg testing is simple to implement, not expensive and may still be useful in resource poor settings to identify those women at increased risk of transmitting HBV leading to viral persistence in their offspring.

Sequence analysis indicated the presence of codon changes across the HBsAg, precore and basal core promoter regions. The high prevalence of e-null viruses bearing pre-core/core and basal core promoter mutations may indicate a propensity for early progression to serious liver disease in the future [29] and those making health care predictions should bear this in mind. No HBV primary resistance mutations were seen probably reflecting the fact that very few women were on antiretroviral therapy, with antiviral drug residues detected in only three out of 50 samples.

Variation in the *HBsAg* sequence in persistent HBV infections was notable and seems phenotypically more common in the HIV co-infected host. There was no instance of major epitope loss as can be found in the classic vaccine escape G145R variant [14,30]. However, the possible rescue by parturition of viruses bearing altered coding and expression of HBsAg derived from HIV-infected mothers whose viruses are beginning to escape host immune surveillance clearly exists. Whether drug regimes where lamivudine is the sole HBV-active agent which results in a significant risk of developing HBV resistance [31] could also potentiate the emergence of the vaccine escape-like viruses [32,33] remains to be seen. In this study surprisingly few women were on antiretroviral therapy, in particular few were on agents active against HBV, probably reflecting poor roll-out of antiretroviral therapy at the time of the study. Nevertheless the possible infant rescue of viruses bearing altered HBsAg lends urgency to the need to alter the timing of HBV immunisation and the espousal of the more potent anti-HBV drugs like tenofovir and entecavir for the treatment of those women who are HBeAg seropositive in the future.

There is one important limitation of this study which must be considered when extrapolating from these data. The women recruited to this study were only those who presented to antenatal clinics for booking and a significant part of the pregnant population would have been excluded from this study through not attending clinic. The reasons for poor access to health care are multiple and range from financial restraints, lack of understanding of the benefits of early booking to poor provision of health care services in some areas. Those pregnant women who do not access antenatal clinics are therefore more likely to have poorer access to health care and potentially to have more advanced HIV disease. In these women the loss of control of HBV is likely to be more profound and they may represent a reservoir of mutated HBV variants. However, no immune status data in the HIV-infected women was collected nor was there any way to establish the timing of their HIV infection.

This study indicates that HIV-infected pregnant women, who may remain relatively well and immune-competent, nevertheless have evidence of HBV escape when compared to HIV-uninfected women. One in six HBV-infected pregnant women, irrespective of HIV status, is HBeAg seropositive and likely to transmit HBV to their offspring. These data support the call for a shift of HBV immunisation closer to the time of birth or, where this is deemed impractical consideration should be given to the addition of a birth dose of HBV vaccine to the current schedule. The data highlight the need for further studies to determine the risk and virological outcome of vertical transmission from mothers in this population.

## Figures and Tables

**Table 1:** Basic demographic data for the age- and sex-matched cohorts of HIV-infected (*n* = 1543) and HIV-uninfected (*n* = 1546) antenatal clinic attendees from the Western Cape.

	HIV-uninfected (*n* = 1546)	HIV-infected (*n* = 1543)
Age	Median 26 yrs	Median 26.8 yrs
	(IQR 23,31)	(IQR 23,31)
	(Range 12–44)	(Range 12–44)
Black	1297 (83.9%)	1323 (85.7%)
Coloured	203 (13.1%)	179 (11.6%)
Other/unknown	46 (3.0%)	41 (2.7%)

Education ≤ gd10^a^	998 (64.6%)	886 (57.4%)

Parity	Median 1	Median 1
	(IQR 0,2)	(IQR 0,2)
	(Range 0–7)	(Range 0–7)

a Secondary school.

**Table 2:** HBV serology and HBV DNA levels in samples from HIV-uninfected and HIV-infected antenatal clinic attendees.

	HIV-uninfected	HIV-infected	*p*-Value
HBsAg positive^a^ (%)	44/1546 (2.9)	53/1543 (3.4)	0.404
HBeAg positive, AntiHBe negative (%)	7^c^/41^b^ (15.9)	10/53 (18.9)	0.872
HBeAg negative, Anti-HBe positive (%)	32^c^/41^b^ (72.7)	43/53 (81.1)	0.912
HBV DNA >10 IU/ml (%)	34/41 (77.2)	44/53 (83.0)	1.000

a Confirmed by neutralisation.

b Three HBsAg-positive samples had insufficient volumes left for further testing.

c One sample was negative for both HBeAg and anti-HBe and another one positive for both HBeAg and anti-HBe.
